# Effects of the application techniques of self-adhesive resin cements on the interfacial integrity and bond strength of fiber posts to dentin

**DOI:** 10.1590/1678-775720150600

**Published:** 2016

**Authors:** Ana Paula Ribeiro do Vale Pedreira, Paulo Henrique Perlatti D'Alpino, Patrícia Nóbrega Rodrigues Pereira, Sasha Braun Chaves, Linda Wang, Leandro Hilgert, Fernanda Cristina Pimentel Garcia

**Affiliations:** 1 Universidade Católica de Brasília, Departamento de Odontologia, Campus I, Brasília, DF, Brasil; 2 Universidade Anhanguera de São Paulo, Curso de Odontologia, Grupo de Pesquisa em Biomateriais em Odontologia, São Paulo, SP, Brasil; 3 Universidade de Brasília, Faculdade de Ciências da Saúde, Departamento de Odontologia, Campus Universitário Darcy Ribeiro, Brasília, DF, Brasil; 4 Universidade de Brasília, Faculdade de Ciências da Saúde, Instituto de Biologia, Departamento de Genética e Morfologia, Campus Universitário Darcy Ribeiro, Brasília, DF, Brasil; 5 Universidade de São Paulo, Faculdade de Odontologia de Bauru, Departamento de Dentística, Endodontia e Materiais Odontológicos, Bauru, SP, Brasil

**Keywords:** Compressive strength, Post and core technique, Resin cements, X-ray microtomography

## Abstract

**Objective:**

To evaluate the influence of an application technique of a glass-fiber post using self-adhesive resin cements on the push-out bond strength and the presence of bubbles in the root thirds. The cements were either applied according to the manufacturer's instruction or using a commercial delivering system (Centrix), at which the cement pastes were collected and applied after manipulation.

**Material and Methods:**

Self-adhesive resin cements (RelyX U200/3M ESPE-U200; Maxcem Elite/Kerr-MAX; Clearfil SA Cement/Kuraray-CSA) and a conventional cement (RelyX ARC/3M ESPE-ARC) were used to cement a post and applied either based on the manufacturer's instructions or using a Centrix syringe to deliver the cements directly onto the post of choice, or directly into canal. The roots were scanned with a micro-computed tomography (μCT) and then sectioned into nine 1-mm thick slices for a push-out bond strength test. The μCT images showed the percentage of bubbles in the root thirds (cervical, medium, and apical). Data were analyzed with three-way ANOVA/Tukey (α=0.05).

**Results:**

Triple interaction was not significant (p>0.05). The interaction “material” vs “root third” was not significant. A significant interaction was observed between “material” vs “application technique” (p<0.05). For ARC, U200, and MAX, significantly lower percentages of bubbles were observed when the Centrix syringe delivered the cements. Equivalent percentages of voids were observed for CSA, irrespective of the application technique (p>0.05). Significantly higher bond strength was observed when the self-adhesive resin cements were applied using the Centrix delivery system, in comparison with the manufacturer's instructions (p<0.05). Bond strength varied with the root third: cervical>medium>apical (p<0.05). No correlations were found between the bond strength and voids.

**Conclusions:**

Bond strength and voids are negatively influenced by the conventional application technique for luting fiber posts. The delivery system (Centrix) seems to produce better results when cementing fiber posts.

## INTRODUCTION

The restoration of endodontically treated teeth has evolved in the last two decades from an empirical approach to the application of biomechanical concepts based on scientific evidence^24^. Within this context, the preservation of tooth structure, the presence of ferrule effect, and the use of adhesive materials for cementation are among the most important requirements for long-term success of this type of restoration^8^.

Glass-fiber reinforced resin posts have shown successful long-term clinical survival^7^. The clinical effectiveness of these restorations has been attributed to their biomimetic behavior similar to dentin^1^. In addition, fiber posts passively adapt to the root canal walls. Thus, the effectiveness of the bonding procedures for luting these posts plays an important role on the clinical performance of these restorations^9^. Resin cements have been generally recommended for luting of resin posts. However, this procedure is considered sensitive because of its many operatory steps. Debonding and leakage have been pointed out as main causes of fiber post failure^14^. The failure potential of a cemented restoration under applied forces is related to the mechanical properties of the individual parts, and flexural strength and elastic modulus are important properties regarding the ability of the cement to resist stress without fracture and/or permanent deformation^23^.

Resin cements are usually applied to cement posts in thin layers. A homogenous and an adequate thickness of resin cement is a prerequisite for an improved retention. Post adaptation is of paramount importance, especially at the coronal root third, at which the cement layer is virtually thicker, and bubbles are likely to occur^29^. These factors may also lead to post debonding and restoration failure. Thus, the thicknesses of the resin cement at different root canal levels surrounding the post needs to be evaluated. Improved delivery systems have recently been developed in order to mix and provide, according to the manufacturers, a consistent bubble-free paste-paste mixture. These systems consist of a syringe that extrudes the ready-mixed cement through mixing tips directly into the root canal. Hence, the choice of the post system, the bonding technique, the characteristics of dentine substrate, and the luting agent used and its application technique to cement the post are determinants for the performance of the final restoration^28^.

The main objective of the introduction of self-adhesive resin cements was to overcome the drawbacks of other types of cements used to cement indirect restorations to tooth preparations^21^. This category of materials requires no acid etching, priming, or bonding, claimed to be technique-sensitive steps allowing the formation of secondary reactions between the self-adhesive resin and hydroxyapatite by means of chemical bonds^4^. This bonding mechanism represents an important characteristic when compared with other resin cements, which are micromechanically bonded to the dental tissues^26^.

Drawing upon two hypotheses, this study investigated the influence of the application technique used to cement a fiber post to the root canal on the push-out bond strength, and on the presence of bubbles in the cement layer surrounding the post in the different root thirds, when evaluating different self-adhesive resin cements. The following research hypotheses were tested: (i) the application techniques of the resin cements do not affect the presence of bubbles in the different root thirds, irrespective of the material tested; and (ii) different application techniques do not affect the push-out bond strength, irrespective of the cement tested and the root thirds.

## MATERIAL AND METHODS

### Experimental design

**Figure 1:**
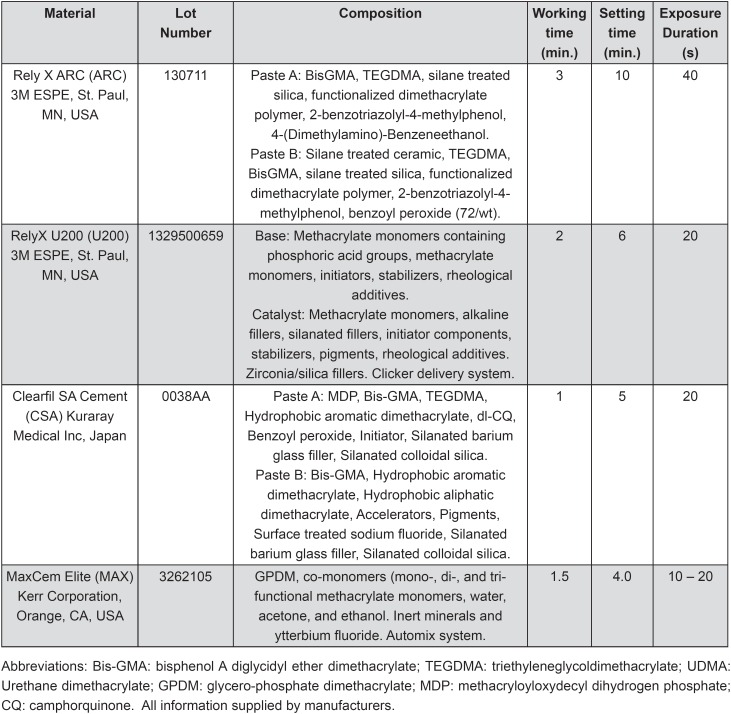
Composition, manufacturers, and batch numbers of the cements used

In this *in vitro* study, the push-out bond strength of a glass-fiber post to root dentin and the presence of gaps/voids in the layer formed surrounding the posts using different cements were evaluated as a function of the application technique of luting cement, according to the following factors: (1) resin cements at four levels: RelyX ARC (ARC), RelyX U200 (U200), MaxCem Elite (MAX), and Clearfil SA Cement (CSA); and (2) application techniques (at two levels), and root third (at three levels). The characteristics of the cements selected are described in Figure 1. Twenty four groups were categorized and treated according to the experimental design.

### Specimen preparations

Forty sound recently extracted human premolars were scaled, cleaned with slurry of pumice and water, and stored in a 0.1 % Thymol solution at room temperature. Teeth were obtained according to guidelines established and approved by the Human Assurance Committee (file no. #05411). The crowns were then sectioned below the cement-enamel junction in order to obtain 15-mm-long roots. Endodontic access was achieved, with the working length established at 14 mm. Cleaning and shaping was performed using a crown-down canal root preparation technique employing nickel-titanium rotary instruments (Dentsply Maillefer, Ballaigues, VD, Switzerland) to size 30 and 0.09 taper. To remove tissue remnants during instrumentation, the canals were repeatedly irrigated using 2% NaOCl after each instrumentation step. At the end of the preparation, 3 mL of 17% EDTA solution (Vista dental products, Inter-med Inc., Racine, WI, USA) was delivered into the root, and the solution was left in place for 3 min before flushing with 2% NaOCl. Then, the canals were flushed by rinsing with distilled water to remove remnants of chemicals. Root canals were briefly blotted with taper paper points.

### Root canal filling

All specimens were prepared and filled by a single operator. Root canals were filled with gutta-percha and a calcium hydroxide based sealer (Sealer 26, Dentsply, Rio de Janeiro, RJ, Brazil) using lateral condensation technique. Subsequently, the crown sides of all roots in both groups were cleaned from material remnants. The coronal portion of filled roots was temporarily sealed with glass ionomer cement (Vitremer, 3M ESPE, St. Paul, MN, USA) and stored in 100% humidity at 37°C for 24 h. After storage, the sealing material was removed using a #245 carbide bur (Brasseler, Savannah, GA, USA) in a high-speed handpiece. Then, the root canal was enlarged using a Gates drill (size 2) and a low-speed drill provided by the manufacturer of the post-system (Reforpost, size 3, Angelus, Londrina, PR, Brazil). The depth of the post-space preparation was 10 mm from the CEJ, and the diameter was kept constant for all teeth (1.5 mm), resulting in 3 mm of apical sealing. After preparation, the root canals were cleaned with distilled water, gently dried with absorbent paper points, and randomly assigned into four experimental groups (n=10), according to the luting cement used to cement the posts (Figure 1).

### Post cementation procedures

**Figure 2:**
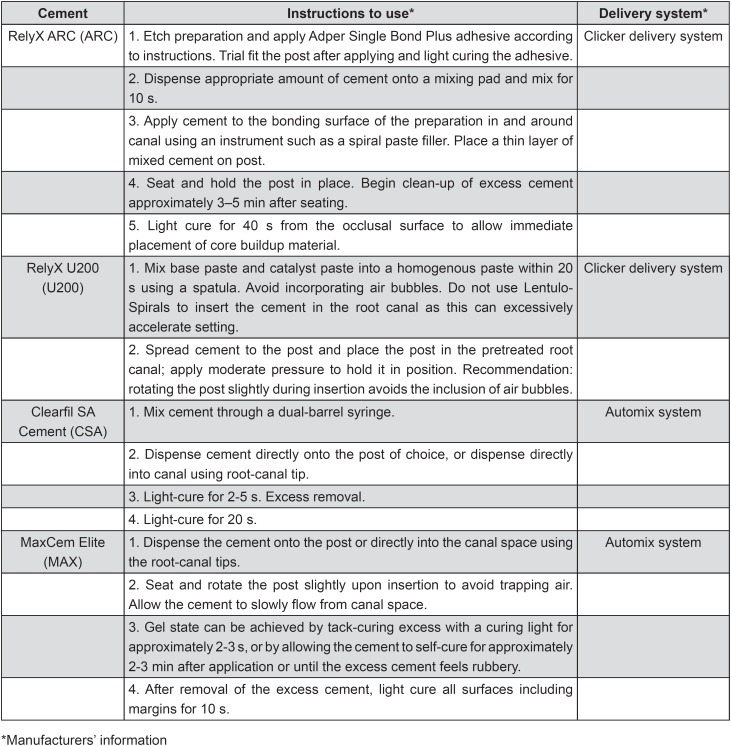
Application modes of the cements investigated

All of the roots received a glass fiber post 1.5 mm in diameter (Reforpost, Angelus, Londrina, PR, Brazil). Before cementation, the posts were placed into the root canal to confirm the position and adaptation, according to the manufacturer's instructions. Then, the posts were cleaned with 70% ethanol, dried with absorbent paper towels, and silanized (Silane Angelus, Londrina, PR, Brazil) for 1 min. The experimental groups were designed according to two subgroups: Group A – application of the luting cements into the root canals according to manufacturer's instructions (Figure 2), or Group B – application of the luting cements using a commercial delivery system (E/Z Syringe with disposable tube and plug system AccuDose Low Viscosity, Centrix, Shelton, CT, USA). For Group B, after manipulation, the cements were placed on a mixing pad and then backloaded into the tube. Then, a plug was inserted into the backside of the tube. The loaded tube was then placed into the syringe barrel. Finally, the syringe was squeezed with slow, steady pressure to deliver the cements according to manufacturer's instructions (directly onto the post of choice, or directly into canal). The roots with cemented posts were entirely submerged in 1.0 mL of deionized water at 37°C for 7 d in Eppendorf containers to allow the sealer to set completely before further analysis. Working time, setting time, and also the light-curing procedures were performed as recommended by the manufacturers (Figure 1). Cements were light-cured with a LED curing light (Bluephase, Ivoclar Vivadent, Schaan, Liechtenstein), with a radiant emittance of 1000 mW/cm^2^.

### Micro-Computed Tomography (μCT) analysis

Previously to the push-out tests, the specimens were mounted on stubs fitting the specimen stage of a μCT scanner (Skyscan 1076, MicroCT Skyscan, Kontich, Belgium). Care was taken to prevent dehydration of the roots during the scanning procedures in the μCT with phosphate buffered saline.

The filled roots were scanned by using a high-resolution micro-CT instrument (SkyScan 1072, MicroCT Skyscan, Kontich, Belgium) at a pixel size of 9.05 mm and an exposure time of 3.1 s. The resulting images were reconstructed by using NRecon software (SkyScan) that produced 2-dimensional (2D) slices of the inner structure of the filled roots. CTAn and CTVol software (DataViewer, Skyscan, Kontich, Belgium) were used for volumetric analysis and to create 3-dimensional (3D) models for the roots. The total volume of the root canal, the volume of bubbles/voids/gaps within the root canal, and the percentage volume of bubbles were measured in each sample.

### Push-out bond strength test

The specimens were attached to the arm of a low-speed machine (Isomet, Buehler, Lake Bluff, IL, USA) with diamond saws and sectioned perpendicularly to the long axis under water cooling to obtain nine 1-mm-thick specimens out of each root: three coronal, three medial, and three apical specimens. The thickness of the slices was measured with a digital caliper (Absolute Digimatic, Mitutoyo, Tokyo, Japan). Each slice was separately identified in Eppendorf containers containing 1 mL of deionized water. The tests were performed in a universal testing machine (MTS, Material Test System 810, Systems Corporation, Eden Prairil, MN, USA) at a cross-head speed of 0.5 mm/min in the apical-coronal direction. Each slice was positioned on the base in such a way that the coronal surface of the slice faced the device, and the post was centered over the opening in the jig. The post-segments were loaded with the punch pin (Ø 0.9-1.1 mm) centered on the post-segment, with no contact with the surrounding dentin surface. The force of post dislocation was registered at the moment of displacement of the post fragment from the canal. The maximum failure load was recorded in Kgf and converted into MPa. The bonding surface area was calculated for each slice using the following formula (1):


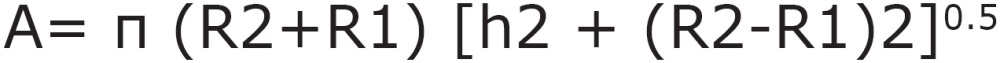


where Π=3.14, R2=fragment coronal radius, R1=fragment apical radius, and h=slice thickness.

### Statistical analysis

Push-out bond strength and bubble/voids test data were analyzed using three-way ANOVA (factors: cements, application techniques, and root thirds) and Tukey's *post hoc* multiple comparison test. All statistical testing was performed at a preset alpha of 0.05.

## RESULTS

**Figure 3:**
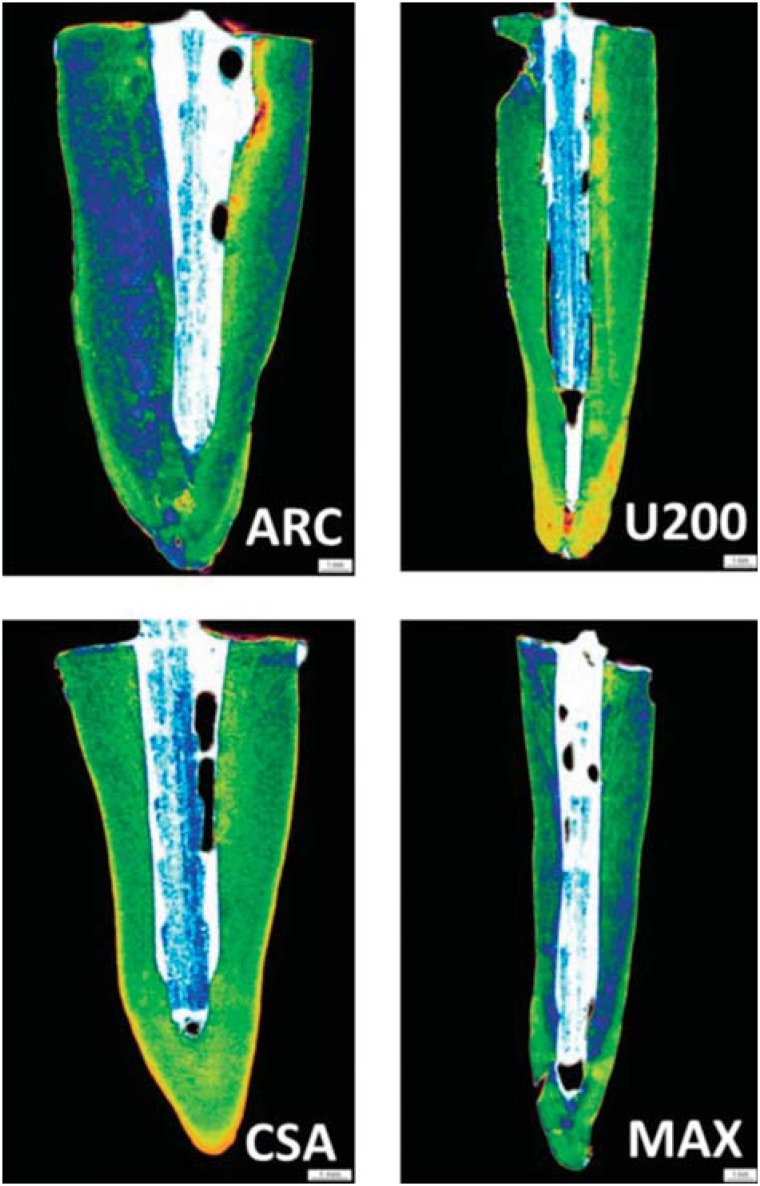
μCT scanning of the roots cemented using the resin cements ARC (ARC), RelyX U200 (U200), Clearfil SA Bond (CSA), and Maxcem Elite (MAX) as a function of the manufacturer's instructions. According to the results, the percentage of bubbles, voids, and gaps was higher in the groups where the posts were cemented according to the manufacturer's instructions

**Figure 4:**
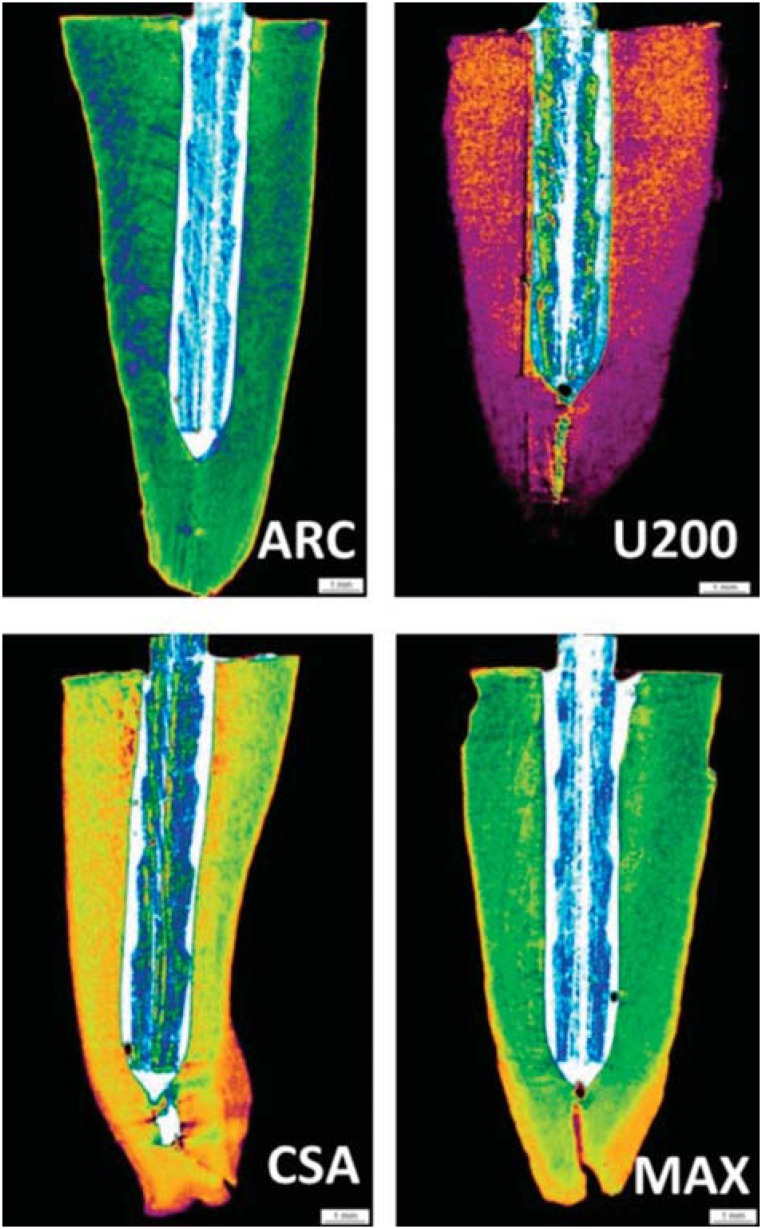
μCT scanning of the roots cemented using the resin cements ARC (ARCA), RelyX U200 (U200), Clearfil SA Bond (CSA), and Maxcem Elite (MAX). The percentage of bubbles, voids, and gaps was lower using a commercial delivery syringe (Centrix) to apply the cement into the root canal

Illustrative images of the interfacial area surrounding the post obtained in the μCT analysis are depicted in Figures 3 (A to D) and 4 (A to D). The morphological analysis demonstrated that groups B, in which a delivery system was used to insert the cements into the root canals, exhibited lower percentages of gaps, compared with groups A. The calculated percentages of voids/gaps at the interfacial cement layers adjacent to the posts as well as the statistical analysis are displayed in Table 1. The statistical analysis demonstrated that the triple interaction among the factors was not significant (p>0.05). In addition, the factor root third was not significant. The interaction “material” and “application technique” was significant (p<0.05). When the results of groups A and B were compared, the luting cement CSA presented equivalent results in terms of gaps/voids at the interface, irrespective of the application technique (p>0.05). ARC, U200, and MAX exhibited significantly lower percentages of bubbles when the cements were applied using the Centrix delivery system (p<0.05).

**Table 1:** Percentage of voids/gaps *per* cement according to application technique

	Manufacturer's instructions	Application using Centrix syringe
ARC	3.5 (2.0)^bA^	0.7 (1.1)^bB^
U200	7.4 (3.9)^aA^	0.9 (1.5)^abB^
MAX	3.6 (2.4)^bA^	0.4 (0.4)^bB^
CSA	3.8 (2.4)^bA^	3.2 (3.9)^aA^

In parenthesis: standard deviation (n=5)

Different letters, uppercase for columns, lowercase for rows: significant (p<0.05)

The results of the push-out bond strength test are displayed in Table 2. Triple interaction was not significant (p>0.05). The interaction “material” vs “root third” was not significant. A significant interaction was observed between “material” vs “application technique” (p<0.05). Significantly higher bond strength means were provided when the cement CSA was applied using the Centrix syringe, irrespective of the root third (p<0.05). RelyX ARC presented significantly lower bond strength means compared with other cements, irrespective of the root third and application technique (p<0.05). MAX and U200 exhibited intermediary bond strength means when applied with Centrix syringe, significantly lower than that of CSA (p<0.05). Significantly higher bond strength means were observed in the experimental groups in which the Centrix syringe was used to apply the self-adhesive resin cements U200, MAX, and CSA (p<0.05). For ARC, no significance was observed when the application techniques were compared, irrespective of the root third (p>0.05). In addition, the variable root third was significant and followed the sequence in terms of push-out bond strength means: cervical (18.4 MPa)>medium (14.6 MPa)>apical (10.9 MPa) (p<0.05).

**Table 2:** Push out bond strength (MPa) means according to resin cement and application techniqu**e**

	Manufacturer's instructions	Application using Centrix syringe
ARC	7.1 (2.0)^a,A^	10.6 (4.3)^a,C^
U200	12.7 (2.5)^a,B^	19.9 (4.0)^b,B^
MAX	11.1 (3.5)^a,A^	20.2 (3.9)^b,B^
CSA	9.7 (2.4)^a,A^	29.7 (2.4)^b,A^

In parenthesis: standard deviation (n=5)

Different letters, uppercase for columns, lowercase for rows: significant (p<0.05)

**Figure 5:**
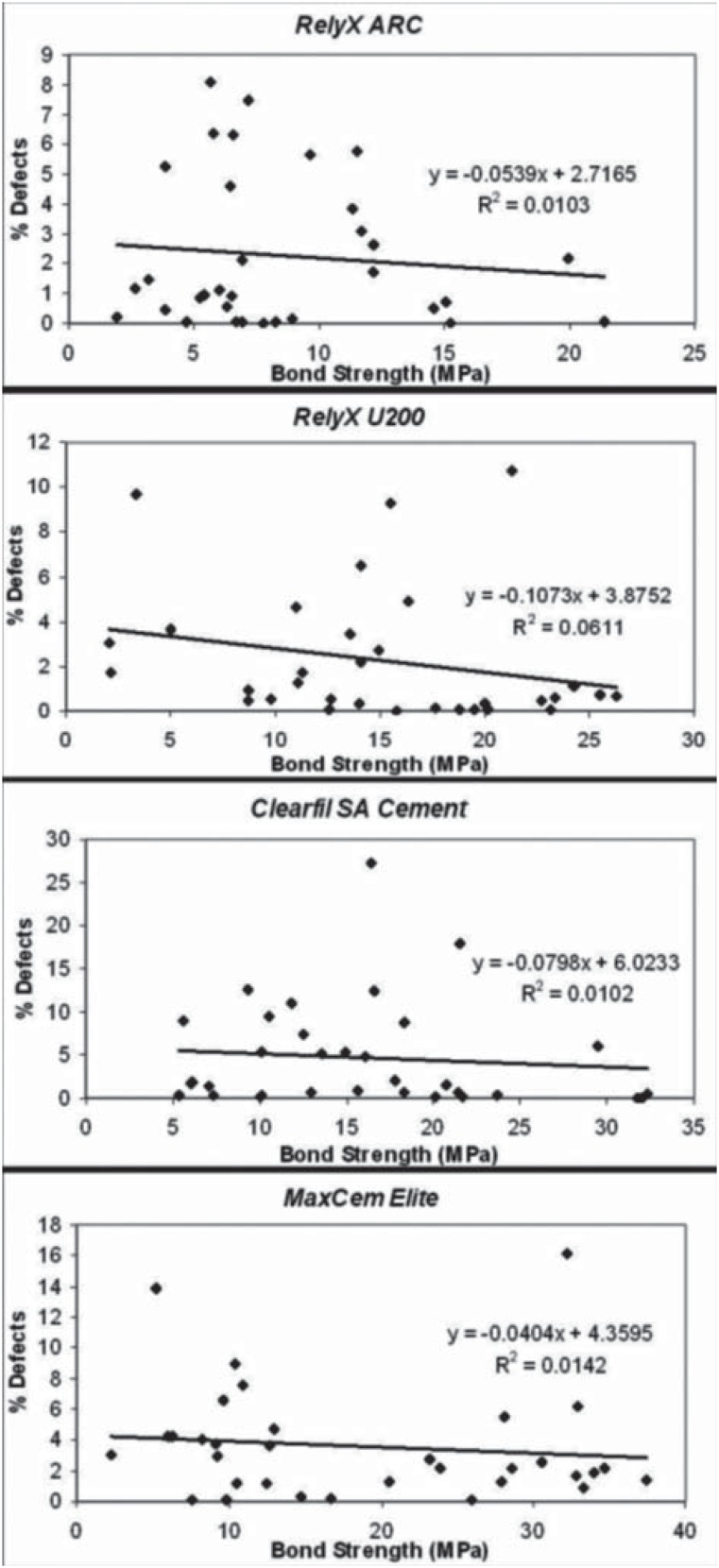
Regression analysis of percentage of interfacial defects vs bond strength for all resin cements tested

Regression analysis was performed on the plot of push-out bond strength vs percentage of bubbles (gaps/voids) for all luting cements tested. Correlations between the bond strength and presence of gaps/voids were found varying from “very weak negative” to “moderate negative” (Figure 5). It can be inferred that the push-out bond strength was not impacted by the presence of gaps/ voids at the interfacial cement layer, irrespective of the application technique and luting cement.

## DISCUSSION

The application of the resin cements into the root canals is regarded to be one of the main steps to guarantee an improved bonding to both the post and the dentinal tissue walls. Post type, diameter, and cement thickness are also claimed to be some of the factors that may affect the retention of post systems^15^. Regarding the influence of the thickness of interfacial cement layer, there is no consensus concerning the optimal thickness of the resin cement interface to optimize post retention^22^. Bubbles and artifacts present at the cement/post interface can negatively influence the performance of adhesive restorations, predisposing to infiltration and decreasing its durability. The presence of bubbles or voids represent areas of weakness within the material and is thought to occur in a lower percentage in thin, uniform cement layers^11^. In a previous study^29^, the authors compared the presence of bubbles when different commercial cements were applied for luting an endodontic post using either the conventional application technique or an automix dispensing tip (called “flexible root-canal-shaped application aid”). A larger number of voids and bubbles occurred when the conventional technique was used in comparison with the automix dispensing application, which allowed a more homogenous cement interface for the self-adhesive luting materials tested. In the present study, for both RelyX products, the cements were dispensed onto a mixing pad and mixed into a homogenous paste according to manufacturer's instructions. Then, the cement was spread to the post and placed in the pretreated root canal. For MAX and CSA, the cements were dispensed using an automix dispensing device directly onto the post, or directly into the canal using a root-canal tip. According to the findings of the study, except for CSA, which exhibited similar results using both application techniques, all resin cements presented significantly lower percentages of gaps/voids when the Centrix syringe was used to deliver the materials into the root canal to cement the posts. In other words, applying CSA using the Centrix syringe had no influence on the percentage of bubbles at the interface. Considering that the application technique reduced the presence of bubbles in most of the cements tested, the first research hypothesis that the application techniques of the resin cements do not affect the presence of bubbles in the different root thirds, irrespective of the material tested, was not accepted.

In a previous study^22^, areas and volumes of post, cement, and voids/bubbles were evaluated in the post space of oval-shaped premolars restored either with oval or circular posts using μCT. The authors identified significantly higher volumes of bubbles in the cement at the cervical third of the root canal, the region at which higher bond strengths are generally found. The authors speculated that the presence of bubbles would not represent “weak points” in the material resistance, but the opposite, acting as stress relief regions within the material. A great number of bubbles was observed in the A groups (manufacturer), irrespective of the root third. It can be speculated that voids and bubbles were included within the luting materials during the mixing step and/or application into the root canal. An incomplete mixing of the paste/paste components or a cement viscosity unsuitable for luting inside the root canal can be responsible for the development of these defects^25^. This aspect was minimized in the groups B (excepted for CSA), in which after appropriate mixing procedures, the cements were subsequently applied using the Centrix syringe delivering system.

The results of the present study indicated, regarding the push-out bond strength test, that applying the cements into the root canal using a commercial delivery system after base/catalyst pastes mixtures provided significantly higher means for the self-adhesive resin cements. For ARC, no significance was observed when the bond strength means of both application methods were compared. It was also found that the bond strength decreased towards the apical direction, and it may be due to several factors, including the numerous variables involved in root canal bonding technique, such as humidity control, solvent evaporation, presence of remaining chemical cleaning agents, access to light inside the root canal, and C-factor, among others^20^. Thus, the second research hypothesis, which anticipated that the different application techniques do not affect the push-out bond strength, irrespective of the cement tested and the root thirds, was not accepted.

Reasons that explain the improved bond strength for the self-adhesive resin cements U200, MAX, and CSA rely on the fact that the application method using a commercial delivery system (Centrix syringe) may have provided an improved monomer dentinal tissue interaction. This may be due to a decrease in the cement viscosity that allows the monomer/comonomer systems to enhance the diffusion of the reacting species, leading to an increased rate of reticulation, especially in the initial stages of polymerization^2^. It has been also claimed that the ability of the self-adhesive resin cements to diffuse and decalcify the underlying dentin effectively is related to the increasing viscosity due to an acid-based reaction that occurs after paste-to-paste mixing^18^. This was particularly significant for the self-adhesive cements because these materials depend on greater contact with dental tissues to react with hydroxyapatite, allowing a better monomer dentinal interaction with the dental tissues, and enhancing the sealing potential for the prevention of nanoleakage, and possibly extended bonding longevity^5^. In addition, there is a simultaneous neutralization effect that occurs during setting, since there are chemical reactions involving water release and alkaline filler that might help to increase the pH level. In other words, the significant increase in the bond strength observed for the self-adhesive resin cements may be due to the differences in the viscosities when delivering the resin cements and after the settings reactions.

The Centrix delivery system may have also helped to increase the chemical bonding potential of 10-MDP to hydroxyapatite found in CSA cement, which is found to be significantly stronger than that of cements containing the monomer 4-MET^30^. Thus, the mean bond strength when CSA was used to cement the post increased from 9.7 to 29.7 MPa. 10-MDP is also claimed to establish a more stable bonding that reflects in the higher bond strength to dentin of the 10-MDP-based Clearfil SA Cement^12^. The same increase was observed for the self-adhesive resin cement U200 that contains methacrylated phosphoric esters and for the containing glycero-phosphate dimethacrylate MAX.

Self-adhesive resin cements contain multifunctional methacrylate monomers that are ionized at the time of mixing, reacting with the hydroxyapatite mineral portion of tooth tissue in order to promote adhesion^24^. According to the manufacturer's information, MAX also contains acidic monomers and glycerol dimethacrylate dihydrogen phosphate (GPDM), responsible for the bonding mechanism of this self-adhesive cement to dentin. The lower technique sensitivity, because of the elimination of the etching step, is probably responsible for the performance of these materials in the intra-root environment. Among the factors that possibly influence the ability of self-adhesive cements to interact with the substrate are chemical composition, viscosity, and pH. MAX cement tends to maintain its low pH (2.2), whereas for U200 pH increases in 24 h (from 2.8 to 7.0). Some authors suggest that the maintenance of a low pH could have an adverse effect on the bond strength of self-adhesive cements to root dentin^24^.

Reasons that explain the significantly lower bond strength means when the post was cemented with ARC, regardless of the application method, are related to bonding procedures. RelyX ARC is classified as conventional resin cement, associated with a two-step, total-etch conventional adhesive system (3M ESPE, Adper Single Bond 2, St. Paul, MN, USA). After the cement is mixed, its application is performed with numerous clinical steps, in a procedure similar to the application of conventional water-based cements^16^. The use of self-adhesive resin cements to join indirect restorations to tooth preparations is facilitated. In addition, the adhesion strategies employed with self-adhesive resin cements also allow the formation of secondary reactions between the self-adhesive resin and hydroxyapatite, forming chemical bonds^4^. This innovative bonding mechanism represents an important characteristic when compared with other resin cements, which are essentially micromechanical in nature^26^.

The fact that there is a trend of a decreased bond strength as a function of coronal-apical direction can be explained by an inability of the dual-cured cements to reach a similar degree of conversion in the total extension of the cement layer surrounding the posts^13^. This somehow demonstrated a dependence on the photoactivation of the cements^3^, in which the curing light is unable to reach the apical areas. The failure potential of a cemented restoration under applied forces is related to the mechanical properties of the individual components found in the compositions, which is regarded to allow the ability of the cement to resist stress without fracture and/or permanent deformation^23^. Another factor to explain the lower bond strength at the apical region is related to the different tubular anatomy in the apical trend. One of the factors responsible for the different bond strength values at the various depths of the root canal is the ability of the materials to acid etch the walls of the root canals in the case of the conventional ARC cement or for the acidic functional monomer in self-adhesive resin cements. In a previous study it was demonstrated that the etching protocol of the root canal produces different conditioning acid patterns^9^. Apical root dentin is a less favorable bonding substrate because of areas devoid of tubules, irregular secondary dentin, cementum-like tissue on the root canal wall, and numerous accessory canals^17^.

The chemical composition of the self-adhesive resin cements is regarded to present a balanced formulation considering that the polymerization reaction occurs in an acidic environment^6^. The addition of methacrylate monomers that contain phosphoric acid esters simultaneously demineralize and infiltrate both the smear layer and the underlying dentin, providing both micromechanical and chemical bonding^10^. As the pH-neutralization potential has been associated with the mechanical behavior of self-adhesive resin cements over time, materials showing early neutralization are expected to better withstand the mechanical loading that luted posts or restorations are subjected to under clinical conditions^16^. At the same time, it is important that the pH is neutralized in order to avoid impacting the end conversion, considering the effect of both new methacrylate monomers formulation and the technology to initiate polymerization^27^. On the other hand, it is important to mention that there may be an additional glass ionomer-type reaction that occurs at the same time as the free radical polymerization, particularly in the RelyX product^19^.

The use of the Centrix syringe for cement application influenced the bond strength to root dentin of the self-adhesive cements tested. Although significantly lower percentages of bubbles were found when the Centrix syringe was used to apply the cements, no correlations between the presence of bubbles and the bond strength were observed in all of the cements tested. This suggests that the final bond strength of the post to root canal dentin seems to be due to numerous factors such as the physicochemical properties of the resin cement, the application technique, percentage of inorganic fractions and viscosity, wetting ability of cement, conduct diameter and characteristics of the substrate, among others^8^.

This study investigated the influence of the application techniques on the push-out bond strength and on the presence of bubbles at the interfacial area between the post and dentin walls. Although no correlation was found between the bond strength and the percentage of bubbles in the cement layer, it was clearly demonstrated that the factors of resin cement and material application influenced the results, based on the different parameters evaluated. Especially for the self-adhesive resin cements, applying the material using the Centrix delivering system allowed significantly higher bond strength and lower percentage of bubbles at the cement interface in two of these cements. Clinically, restoration longevity depends on the numerous steps before a restorative process is completed. The simplification of clinical steps in using materials is critical to the success of a restorative procedure, as some aspects need to be considered. For the development of future resin cements, *in vitro* and *in vivo* studies are necessary to evaluate the longevity of indirect restorations cemented with this category of resin cement over longer evaluation times.

## CONCLUSIONS

Within the limitations, the following conclusions can be made:

The presence of bubbles at the interfacial cement layer varies as a function of the resin cement and of the application mode, except for CSA cement, which exhibited similar percentages of bubbles in both application techniques tested (hypothesis (i) not accepted);

The bond strength of posts to root canal cemented with self-adhesive resin cements is improved when applied with a commercial delivery system, irrespective of the root third (hypothesis (ii) not accepted);

Based on the parameters evaluated, there is no correlation between the bond strength of post/ cement restorative system to root canal and the presence of bubbles;

It seems that the innovative delivery automix system, claimed to provide better manipulative results, produces similar results in terms of bond strength and bubbles in comparison with that of hand mixing.
